# An Algorithm for Improving Hospital Performance Measures: A Department-centered Approach

**DOI:** 10.5041/RMMJ.10352

**Published:** 2018-10-04

**Authors:** Ziv Gil, Shuli Brammli-Greenberg

**Affiliations:** 1 Department of Otolaryngology Head and Neck Surgery, the Head and Neck Center, Rambam Healthcare Campus, Rapport Institute of Medicine and Research, the Technion–Israel Institute of Technology, Haifa, Israel; 2 School of Public Health, Faculty of Social Welfare and Health Sciences, University of Haifa, Haifa, Israel; 3 Myers-JDC Brookdale Institute, Jerusalem, Israel

**Keywords:** Chair, director, economics, efficiency, hospital, management

## Abstract

In recent years, it has become increasingly important to improve efficiency and economic balance in hospitals. The department chairperson (or Chair) contends with a production function consisting of inputs and outcomes, rife with managerial constraints. These constraints can be reduced with proper management by diverting resources and activity. Lack of a proper management algorithm at the department level is a significant impediment to improving operational efficiency in hospitals without significant additional costs. In this work we aimed to develop and implement a management algorithm in a teaching hospital department, in order to improve performances and quality of care. From September 2012 to December 2017 we developed a novel management algorithm for a surgical department and implemented it in the Head and Neck Surgery Department at Rambam Medical Center, Haifa, Israel. Changes were made to the organization structure and the concept of service provision. We defined core measures reflecting operative actions and outcomes and identified actions that could affect these measures. Based on our analysis of outcomes we constructed a management intervention process that defines operative actions leading to improved performance. The result was over 400% improvement in the department’s outcome measures including quality, activity, and service. Analysis of data from the Israeli Ministry of Health revealed that the department’s ranking in performance measures and volume improved from no. 14 of 23 departments in Israel in 2011, to no. 1 in 2013, which was sustained through 2014–2016. Improvement in efficiency also translated to economic balance and transformation from deficit to profitability. If this algorithm is implemented in the rest of the system, it is expected to improve the function of the hospital as a whole. Our results have the potential to foster the development of a new organizational culture of measurement and improvement in the hospital, and subsequently in the entire health system.

## INTRODUCTION

Most adults have been a hospital or outpatient clinic patient at some time in their life. Many have experienced excessive waiting times, lack of coordination among different departments, unfriendly facilities, and general lack of customer service. While outcome data show that the quality of medical care is improving for most types of illnesses, financial and other fiscal data suggest that this is not the case for operational efficiencies of hospitals.

In recent years, there has been increased awareness of the benefits that successful management of health-care organizations as a whole and hospitals specifically can provide. Hospitals can use their operations and managerial properties to thrive in hostile conditions and to improve organizational performance.^1^

New programs and policies focus on performance measures, quality, and accreditation and have been proven to be an engine of improvement.^2^ Since transparency is a key element for improvement, publishing measures of parallel departments at the same hospital or of different hospitals is of high importance. Such a culture of transparency is executed by the Israeli Ministry of Health and in other OECD countries. Since 2014 the Department of Otolaryngology at Rambam Medical Center (hereinafter, “Rambam”), Haifa, Israel, publishes an annual report of its operational, clinical, and satisfaction measures.^3^ Most programs initiated by the government concentrate on the national level and not directly on the level of the quantal service unit (department, unit, or division). Moreover, most program indicators are clinical and concentrate on quality of treatment rather than performance. At the department level, a methodical management algorithm can support hospital performance by defining core measures, goals, and actions, transforming resources (inputs) into services (outputs). Efficiency at the department level requires achieving the same or higher levels of outputs at the same quality standards, with the same (or fewer) inputs.

Here we show that implementation of such a management algorithm resulted in more than 3-fold growth in the number of elective surgeries, without significant change in manpower resources. Several operative management actions led to more than 300% efficient usage of operation room time, maximizing operational efficiency while minimizing resources.

## METHODS

We have developed a management algorithm based on a teaching hospital department care process. A process is a set of activities and tasks that are performed in sequence to achieve a specific outcome. In the department, a process can be administrative or clinical in nature. A typical departmental process has three phases: *admissions* (and expected admissions) of patients to the hospital; *treatment* of patients (in outpatient clinics or hospitalized); and *discharge* of patients from the hospital. In different departments the weight of each phase may vary. For example, the focus of operative actions in a geriatric department characterized by elderly patients will emphasize the discharge phase, whereas an ophthalmology department that focuses on ambulatory services will dedicate its operative actions to the admission phase. In each of these three phases several specific processes can be addressed to complete department tasks. All specific processes have three components: *inputs*,* transformation*, and* outputs*. In the transformation component, the operative actions of the departmental chairperson or Chair can influence the outcomes. For the purposes of this study the inputs are assumed to be given to the department Chair by the hospital management.

## CONSTRUCTING THE DEPARTMENT MANAGEMENT ALGORITHM

### Inputs and Outputs

Since all specific processes in a department can be broken down into inputs and outputs, these can be tracked to see changes in performance over time. When an internal information system that measures outputs and inputs over time is available and the measurement is balanced and represents the entire picture, outputs can be observed mainly by performance measures.^1^ However, while performance measures should be used strategically to capture a department’s outcomes, in some cases they might fall short. Hence, the management algorithm as defined herein should adopt additional quality monitoring and improvement tools/approaches, such as periodic surveys of patient satisfaction.

### Management Council

A paramount tool that we used in our management algorithm was the *management council*. In order to accomplish all aims of the organization, the department Chair assigned a management council, which took responsibility for review of the inputs and data as well as deciding on interventions and specific operative actions. The overall goal of the council was to eliminate the dominancy of the department Chair and to allow infusion of new and diverse approaches. The goals and composition of the council were according to the following five standards:

1. Discussions focused on gaining an understanding of the important issues
2. A multidisciplinary council composed of three to five people
3. Encouraging freedom to express independent opinions
4. Permitting key members of the department management team to be on the council, but not limiting membership to the department team only
5. Periodic council meetings, at a minimum of once every four weeks

### Establishing Department Management Algorithm

**Figure 1:**
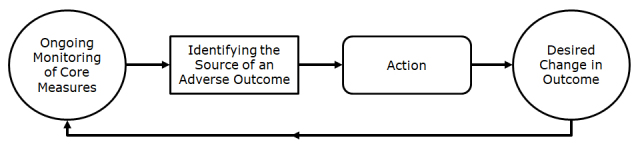
Flowchart of a Management Algorithm.

From the department management perspective, one of the main goals of establishing a management algorithm was to transform the department’s inputs into the department’s outputs in an efficient way. The management algorithm structure is shown in the flowchart in Figure 1.

### Phases of Implementation

#### First phase

To begin with, we determined a set of core performance measures that reliably reflected the activity and outcomes at the department level and which matched the production narrative of the institution. We developed a built-in algorithm for determining which measure could serve as a core measure. This is an innovative concept in the literature related to the management of hospitals in particular and medical service providers in general. It is largely similar to key performance indicators (KPIs) from the business literature of assessing the results of profit-making companies. Key performance indicators are metrics that measure the efficiency and effectiveness of a company. The KPI indices are selected using a series of questions according to the company’s production function and goals.^4^

Our core performance measures were chosen from all the department measures according to the Chair’s objective function. For the Department of Otolaryngology Head and Neck Surgery at Rambam the production function is that of *patient’s primacy*. The meaning of this function is that the core measures should primarily serve the narrative of *patient primacy*. This is similar to the notion of the patient-reported outcomes measures (PROMs) which assess the quality of care delivered to patients from the patient perspective. For example, *average length of stay* (ALOS) and *rate of readmissions* within one week from discharges were chosen as core measures. However, the satisfaction rate of the senior surgeons or overall revenues were not chosen, since they represent the primacy of the senior staff or economic department, respectively, and not patient primacy.

A key feature of a *core measure* is that an action that is constructed to correct an anomaly of function cannot hinder the performance result of a core measure. In other words, while reduction in other counterpart measures is allowed in order to improve function, a decline in the core measures is prohibited, and if it occurs the action should be corrected.

#### Second phase

After observing adverse outcomes of the monitored core measures, the council identified possible parameters that could cause these adverse outcomes. Multivariate analysis was used to identify if changes in core measures were due to external confounders which could not or should not be modified (e.g. patient’s age, comorbidity, type of surgery). This eliminated the possibility of “gaming” (a situation where people use the system to their own advantage by playing with the rules) and manipulation of the data. For example, if the algorithm identified an increase in average length of stay without change in external parameters, the conclusion may be that there was an unnecessary delay in patients’ discharge. The possible actions in this case could include: (1) instruction toward earlier preparation for discharge, (2) refining connections with post-admission care facilities, or (3) interim discharge of patients to a close hotel accommodation. Or if the algorithm identified an increase in waiting time for a new outpatient clinic visit without any effect of external confounders, then the action could include: (1) increasing the number of patient visits per doctor, (2) reserving protected clinic time for new patients, or (3) shuffling personnel from other responsibilities toward strengthening of the activity in the clinic.

#### Third phase

After the index and core measures were identified, the council was assembled to discuss proper action needed to improve the specific measure. The goal was to improve all items that fit the activity of the department. A constraint of a chosen action should be that it should not worsen any core measures. For example, a goal of reducing average length of stay could increase readmission rate. Since such a damage to a core measure is prohibited, two possible actions could be taken: (1) strengthen the relationship with the facilities in the community that absorb patients discharged from the department; and (2) encouraging the medical staff (doctors, social workers, and nurses) to optimize the process of releasing patients to ensure early discharge without putting patients at risk.

An example of a core measures that we aimed to improve was readmission rate within 30 days from discharge. We found that most readmissions were performed by the residents on call. In order to reduce the readmission rate, we instructed the residents to consult with an attending physician regarding the patient they wished to readmit. Using a quasi difference-in-differences analysis, we found that a requirement for an attending approval was associated with a reduction in readmission rate from 3.38% to 1.88%. Such an action describes one process from the analysis stage, to the drawing of conclusions, to an action, through to the result.

### Operative Management Actions

An important part of the management algorithm was choosing the “right” action that will lead to the desired change without worsening the core measures. As defined in the health-care operations management literature,^1^ operative management actions imply a wide use of analytical and optimization tools, as well as extensive use of process and quality improvement techniques, to drive continuous improvement of the performance of a hospital department. Yet, management actions are motivated by the department management function goal.

The management council can promote management practice using operative actions in all three department process phases: *admissions* (and expected admissions) of patients to the hospital, *treatment* of patients (in outpatient clinics or hospitalized), *discharge* of patients from the hospital, as well as some general actions.

Actions should be focused on the core parameters for improving their outcomes. We divided the actions according to the time interval that they were taken: (1) an action that was taken at a specific point of time (a specific date); and (2) an action that was taken through a period of time (days to months).

## DATA AND ANALYSIS

Study population included all inpatient and outpatient cases at the Department of Otolaryngology Head and Neck Surgery at Rambam between the years 2008 and 2017. As reference we observed all in- and outpatient cases at Rambam and respective performance measure outcomes from 2008 onwards. The data were analyzed using the IBM SPSS statistics 21 data analysis and statistical software. All three study datasets were pooled cross-section time series datasets. Pooling cross-sections from different time periods is often an effective way of analyzing the effects of a new intervention or change in policy.

Data were retrieved from the computerized database of Rambam, including information about all patients of the Department of Otolaryngology Head and Neck Surgery who received counseling from the department medical staff or outpatient treatment, or who were hospitalized during the years 2008–2017. All performance measure data for the study period were examined month by month. For actions that were taken at a specific point of time the specific date it was taken was known. Therefore, a simple before-and-after analysis could be made in order to estimate this action’s effect on the relevant core measure.

We performed in-depth interviews with the department Chair and other staff in the department. The interviews were conducted in order to correlate operative management actions to each of the core measures.

The estimated number of elective surgeries in the country was based on the Ministry of Health report of the Israeli national data on all 23 otolaryngology departments in Israel. The data were extrapolated from the number of patients hospitalized in each department, and the percent of elective admissions. Since the only elective admissions in a surgical department were of patients undergoing surgery, we used this number as an “estimated number of elective operations.

## RESULTS

To improve department performance under constrained resources a new management protocol was initiated in the Department of Otolaryngology Head and Neck Surgery at Rambam. Since September 2012, changes have been made and operative management actions were taken to reshape the array of incentives, organization structure, and the concept of service provision.

**Figure 2:**
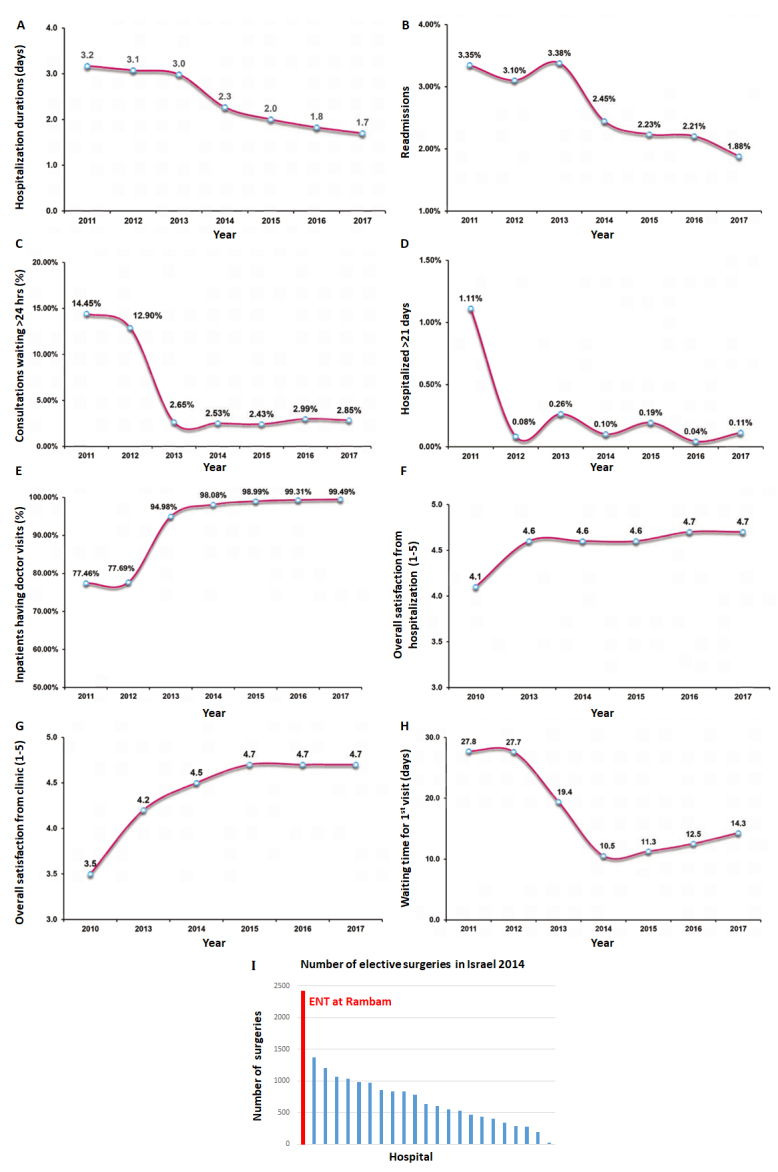
Change in Core Measures during 2011–2017 **A:** Hospitalization duration. **B:** Readmission rate. **C:** Percentage of consultations calls that were not answered within 24 hours. **D:** Percent of patients hospitalized for 21 days or more. **E:** Percentage of patients that have record of doctor visit per day. **F:** Overall satisfaction rate from hospitalization (from 1 to 5). **G:** Overall satisfaction rate from outpatient visits. **H:** Waiting time for first clinic visit. **I:** Distribution of elective surgeries in otolaryngology department in 2014. Number of elective operations (estimated by number of elective hospitalizations) presented for all 23 departments in Israel with Rambam indicated in red (from the Otolaryngology Annual report of Rambam^3^). The number of operations is not considered a core measure.

**Figure 3:**
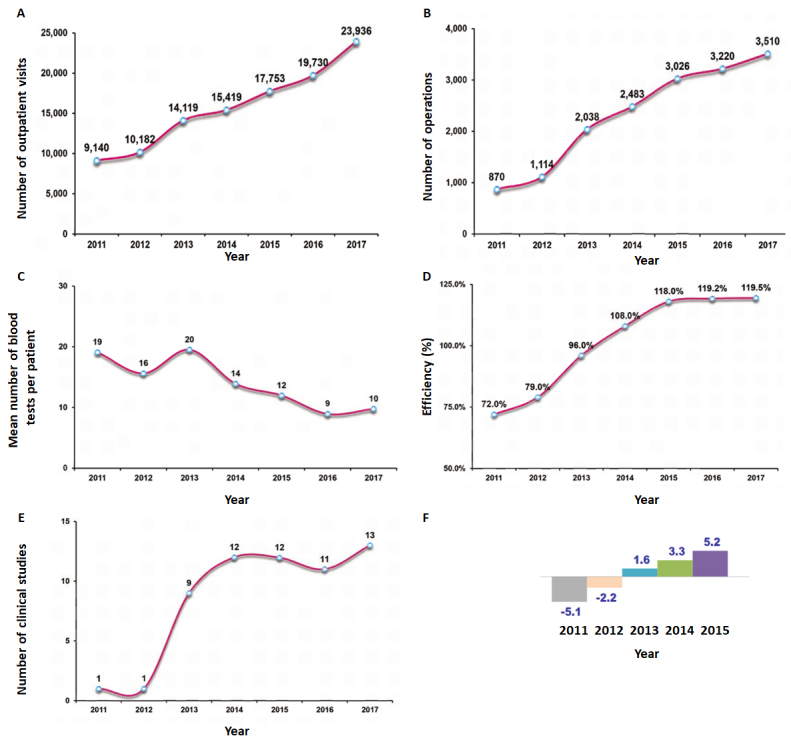
Change in Non-core Measures during 2011–2017 **A:** Number of outpatient visits. **B:** Number of operations. **C:** Mean number of blood tests per patient. **D:** Operating room efficiency. A rate of >100% is achieved when operations extend after 15.00 or when circulation time between patients is <30 min. **E:** Number of clinical studies. **F:** Overall net revenue in millions of New Israeli Shekels (NIS).

In addition to the actionable examples in Table 1, it is important to note that use of assistive technologies such as smartphone apps and information technology systems play a major role in the management algorithm. Hence, technology as well as management action should be considered. As shown in Figure 2, these and other actions have led to significant improvement in the department’s core measures of quality, activity, and service. In addition, Figure 3 shows the change in non-core measures.

**Table 1:** Example for the Type of Actions Taken.

Type	Examples
Action taken at a specific time point	To decrease the waiting time for in-house consultations, the staff member in charge was requested to send a written report every day at 15:00, stating the number of non-completed consultations. To decrease the waiting time of new patients to the outpatient clinic, the schedule was separated into new patients clinics and returning patients clinics, with priority to the former.
Ongoing action taken over a long time period	In order to increase the number of operations per day, a training program was initiated, to improve the efficiency of a set of surgical procedures. In order to increase surgery volume under limited operating room resources, patients undergoing surgery under general anesthesia in the main operating theatre were shuffled to local anesthesia surgery in the day care facility.

Objective data on the case mix show that since implementation of the management algorithm, the department is now taking care of patients that are more complex, older, and are referred from more distant medical centers. Specifically, average age of patients has increased from 40 to 45 years; there has been an 11% (*P*<0.05) increase in more distant referrals; and the more complex procedures being performed include skull base surgeries, oncological operations, and cochlear implantations.

In 2017, the Otolaryngology Head and Neck Surgery Department performed more than 3,500 elective surgeries and had 24,000 outpatient consulting clinic visits, of which more than half were new patients.

With a resource platform equivalent to the mean of similar departments in Israel, Rambam’s Department of Otolaryngology Head and Neck Department achieved twice the elective surgical volume of any other otolaryngology department in Israel.^5^–^7^ Overall, the department elective volume was equal to 22% of the entire country. As a result of this change in efficiency and productivity the department’s *net* revenues (after deductions and discounts for health plans) grew by over 10 million NIS, from −5.1 million NIS in 2011, to +5.2 million NIS in 2015 (Figure 3).

## DISCUSSION

Patient-centeredness is a fundamental tenet of health-care delivery with the principle that care should be designed around the patient’s needs, preferences, circumstances, and well-being.^8^ More than 15 years ago the Institute of Medicine report recognized and defined patient-centered care as “respectful of and responsive to individual patient preferences, needs, and values and ensuring that patient values guide all clinical decisions.”^9^ In Israel, in the last couple of years patient-centered care has received high priority from the Israeli Ministry of Health.

The professional literature emphasizes the importance of patient-centered care to the performance of health systems. There is evidence that patient-centered care improves quality indices, as well as patient satisfaction, self-welfare, and mental health.^10^–^20^ Patient-centered care also contributes to the system at the organization level, where it improves medical staff work satisfaction, reduces costs, and enhances efficiency of health service usage, since it decreases over-, mis-, and under-use of medical services and increases the success rate of diagnosis.^20^–^23^

Unfortunately, in the traditional department-based organizational structure of a university hospital, patients can often be neglected as a result of fragmented systems of care. Specialty-driven, provider-oriented, economically influenced organizations dominated by profit, research, and education missions might promote too little concern for the patient.^24^ At the center of our management algorithm stands the narrative of the *patient’s primacy* – or, in other words, the interest of the patient comes before any other interests, including those of the physicians and organization. The emphasis that the patient must always be a top priority prevents gaming and minimizes conflicts of interest. We show that with this production function the organization can thrive, maximizing productivity, safety, and transformation from deficit to profit. We suggest that an organization should introduce a management practice and policy that serves the narrative of patient primacy.

In its 2013 report, the UK Department of Health summarized this idea by saying that hospitals should move toward making the quality of care as important as the quality of treatment.^25^ This means putting patients first and foremost in any health-care managerial environment. Principles of “Customer First and Foremost” management are common in other industries.^26^,^27^ Health care is primarily a service sector, even though the industry provides mostly intangible or non-physical “goods,” contrasting with physical objects that can be seen or touched. Hospital services primarily deliver care through providers to patients. These services are uniquely knowledge-based and have high levels of (usually anxious) customer interaction.^1^

Measurement is vital to producing a health-care system that achieves remarkable results. Without measurement, clinicians, institutions, patients, and society cannot readily evaluate the value achieved in the health-care system. Measurement has been associated with important improvements in providers’ use of evidence-based strategies and patients’ health outcomes. Perhaps most important, measures have altered the culture of health-care delivery for the better, with a growing acceptance that clinical practice can be objectively assessed and improved.^28^

Indicators have been defined mainly in three ways:^29^ (1) As measures that assess a particular health-care process or outcome; (2) As quantitative measures that can be used to monitor and evaluate the quality of important governance, management, clinical, and support functions that affect patient outcomes; and (3) As measurement tools, screens, or flags that are used as guides to monitor, evaluate, and improve the quality of patient care, clinical support services, and organizational function that affect patient outcomes. Indicators provide a quantitative basis for clinicians, organizations, and planners, aiming to achieve improvement in the processes by which patient care is provided.

In Israel, quality measures were developed later for hospitals than for the community. Indeed, a recent OECD review of quality in Israeli health care noted that “In contrast to primary care, too little is known about the quality of care delivered in hospitals.”^30^ Nonetheless, major progress has been made since. This transfer of attention and efforts opens a window of opportunity to foster the development of an organizational culture of measurement and improvement of the activity and quality of the hospitalization system in Israel.

In Israel, like in most OECD countries, public hospital medical staff (including the inpatient specialists) are publicly employed and salaried.^31^ Their income, *prima facie*, is not affected by the volume, type of activity performed, or case mix treated. Therefore, in principle, their clinical and administrative decisions are independent of economic considerations or incentives. Nevertheless, using operative management actions that change incentives, organization structure, and the concept of service provision, the Chair can influence staff behavior, and bring about changes in department outcomes: in volumes, type of care, and the case mix treated.

Measurements can be maximized with transparence. Indeed, as mentioned above, the Department of Otolaryngology Head and Neck Surgery issues yearly reports of all activity measured; this has been made available on the hospital’s websites since 2014.^3^ The department is the only one in the nation and one of very few in the world that uses transparence to maximize growth. It is also an integral fragment of the perception of patient primacy.

The importance of improving efficiency and work procedures in hospitals has become increasingly recognized in recent years. Our management algorithm is the first one to focus on the basic service units of the teaching hospital (department, unit, division, and service). We show that implementing the management algorithm, to monitor improvements in department outcomes, ultimately led to improvement in the function of the unit and eventually had the potential to improve productivity and profitability of the hospital as a whole.

We are aware of several limitations of this study. First, the structure of the study limited the framework of building the algorithm (including setting its methodology and framework) to one surgical department. Second, the implementation of the algorithm was conducted by a specific management council. Third, the algorithm was structured to answer a specific production function in a surgical department, and its implementation to other disciplines such as internal medicine and pediatrics awaits further modification. In order to validate the utility of the management algorithm in other departments and management teams we are currently conducting an intervention study in the Department of Neurosurgery at Rambam. Further studies are needed to validate its effectiveness in other departments and health organizations. Nevertheless, we believe that the fundamental structure of the managerial concept will remain valid in various disciplines and organizations.

Staff satisfaction is one of the important corner stones that lead to patient satisfaction. In 2014 a team of independent psychologists from the Interdisciplinary Center Herzliya performed a systematic analysis of the staff satisfaction at the Department of Otolaryngology Head and Neck Surgery at Rambam. The results showed that the overall level of good to high satisfaction rate was 82%. This was distributed between disciplines as follows: paramedical staff, 85%; administration, 82%; nursing staff, 76%; and physicians, 75%. This rate is above the median rate of medical staff satisfaction that was recorded in Israel.^32^

We believe that in Israel’s university hospitals the management algorithm could improve performance since their structure and medical staff are similar. A future step will be to develop a management algorithm for internal medicine departments in university and non-university hospitals.

## CONCLUSIONS

While much effort has been expended in identifying single strategies aimed at improving efficiency in health management, there is still a gap in knowledge of which strategies could be most successful in yielding the optimal solution. To the best of our knowledge, our study presents for the first time the concept of change in the organizational culture by introducing the outline of a management algorithm. Although processes designed to increase operational efficiency and outputs exists in other health systems, our study is the first one known to us that formulates a logic algorithm that is based on measurement, action, and improvement, regarding the activity and quality of a unit. Such an algorithm can be implemented in other health organizations, using a council composed of a multidisciplinary team. Establishment of a measurement protocol that maximizes performance serves the narrative of patient primacy and can lead to economic balance and accountable spending. The empirical assessment of the association between specific management actions and performance can guide hospital board members and policymakers on how to strengthen the foundations and stability of the health system, promoting quality and productivity.

## Abbreviations

Chair: chairperson
KPI: key performance indicator
MOH: Israeli Ministry of Health
